# Supplementary material for: YY males of the dioecious plant Mercurialis annua are fully viable but produce largely infertile pollen
Source: New Phytol. 2019 Jul 25;224(3):1394–404. doi: 10.1111/nph.16016 (PMC6852596; doi:10.1111/nph.16016)
Supplement: Supplementary file 1 — Fig. S1 Restriction enzyme assay to distinguish XY and YY individuals. Fig. S2 Likelihood‐ratio tests for progeny sex ratios. Fig. S3 Comparison in phenotypic traits between XY and YY males of the F1 progeny. Fig. S4 Summary of PCA for phenotypic measurements of F1 males from the hormone‐induced experiment. Fig. S5 Summary of PCA for phenotypic measurements of F1 males from the pruning‐induced experiment. Methods S1 Identification between XY and YY males with a restriction enzyme‐based assay based on sex‐specific SNPs. Table S1 Summary of generalised linear model (GLM) comparison XY with YY males in terms of the first four principal components for morphology traits. Table S2 Pollen observation under electron microscopy. [file NPH-224-1394-s001.pdf]

***New Phytologist* Supporting Information**

Article title: YY males of the dioecious plant *Mercurialis annua* are fully viable but produce largely infertile pollen

Authors: Xinji Li, Paris Veltsos, Guillaume Cossard, Jörn Gerchen, and John R. Pannell

Article acceptance date: 10 June 2019

The following Supporting Information is available for this article:

Figure S1 Restriction enzyme assay based on sex-specific SNPs. **(a)** Illustration of the cutting position of the restriction enzymes that only cut the X or the Y allele. **(b)** Illustration of the restriction enzyme assay to distinguish XY and YY individuals.

(a)

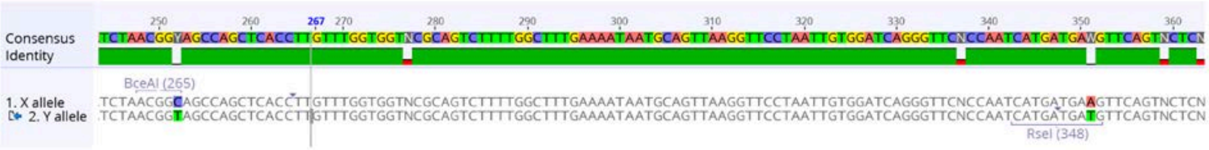

(b)

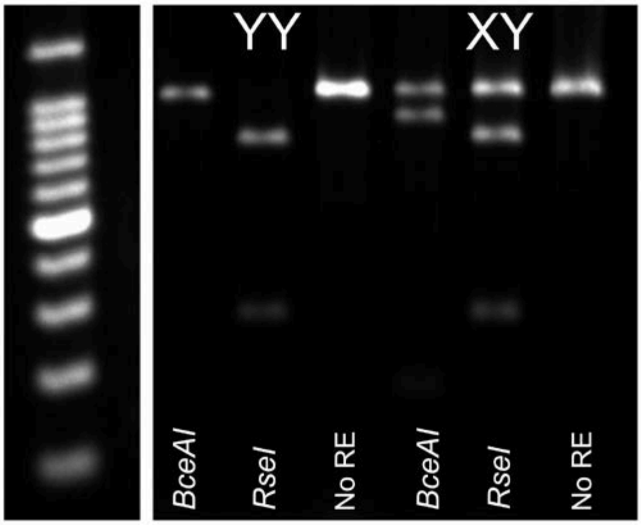

Figure S2 Likelihood ratio tests for progeny sex ratios. **(a)** Likelihood ratio test on the proportion of males among F_1 progeny. p1, equal viability between XY and YY males, resulting in an expected F_1 sex ratio of 0.75; p2, complete YY lethality, resulting in an expected F_1 sex ratio of 0.67. **(b)** Likelihood ratio test on the proportion of males among F_2 progeny. p1, equal fertility between XY and YY males, resulting in an expected F_2 sex ratio of 0.67; p2, complete YY sterility, resulting in an expected F_2 sex ratio of 0.5.

(a)

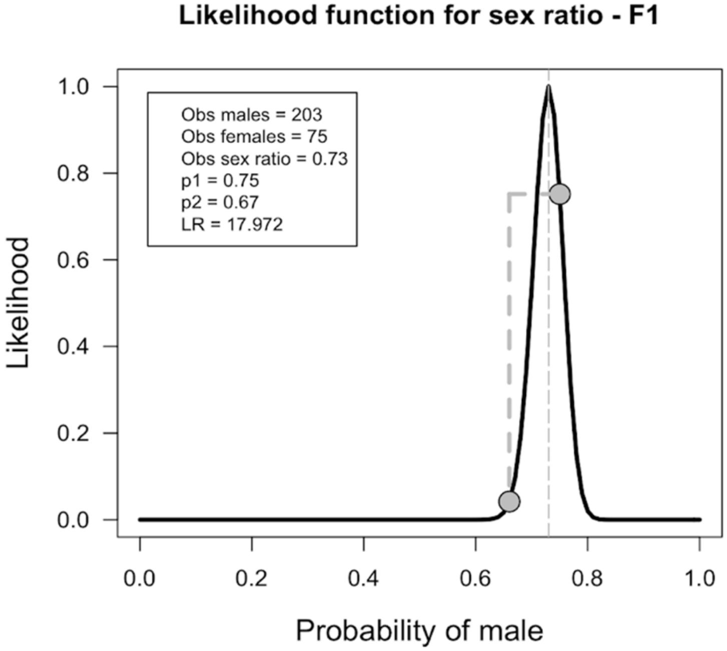

(b)

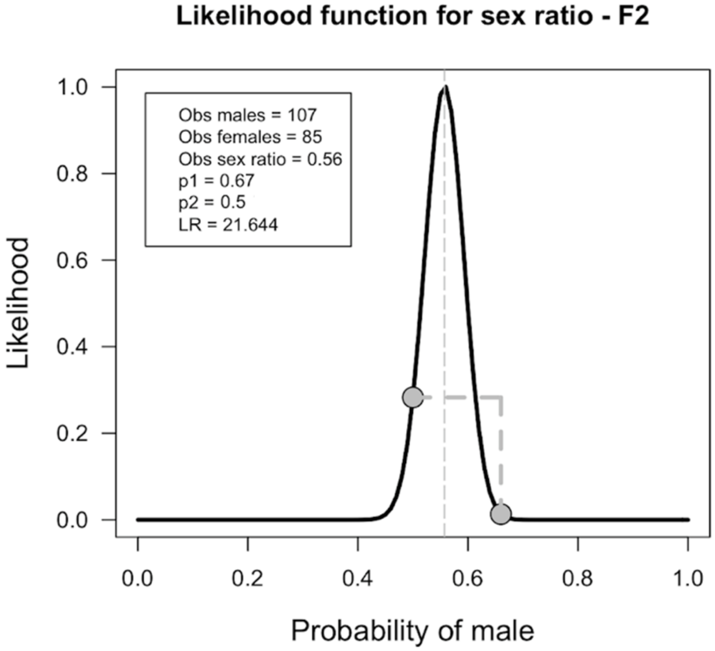

Figure S3 Comparison in phenotypic traits between XY and YY males of the F1 progeny produced by crossing XY plants in the hormone-induced experiment ('hormone') or the pruning-induced experiment ('pruning'). Red asterisk represents the significance level for each trait comparison between XY and YY males in each experiment (* $0.01 < p < 0.05$, ** $0.001 < p < 0.01$, *** $p < 0.001$).

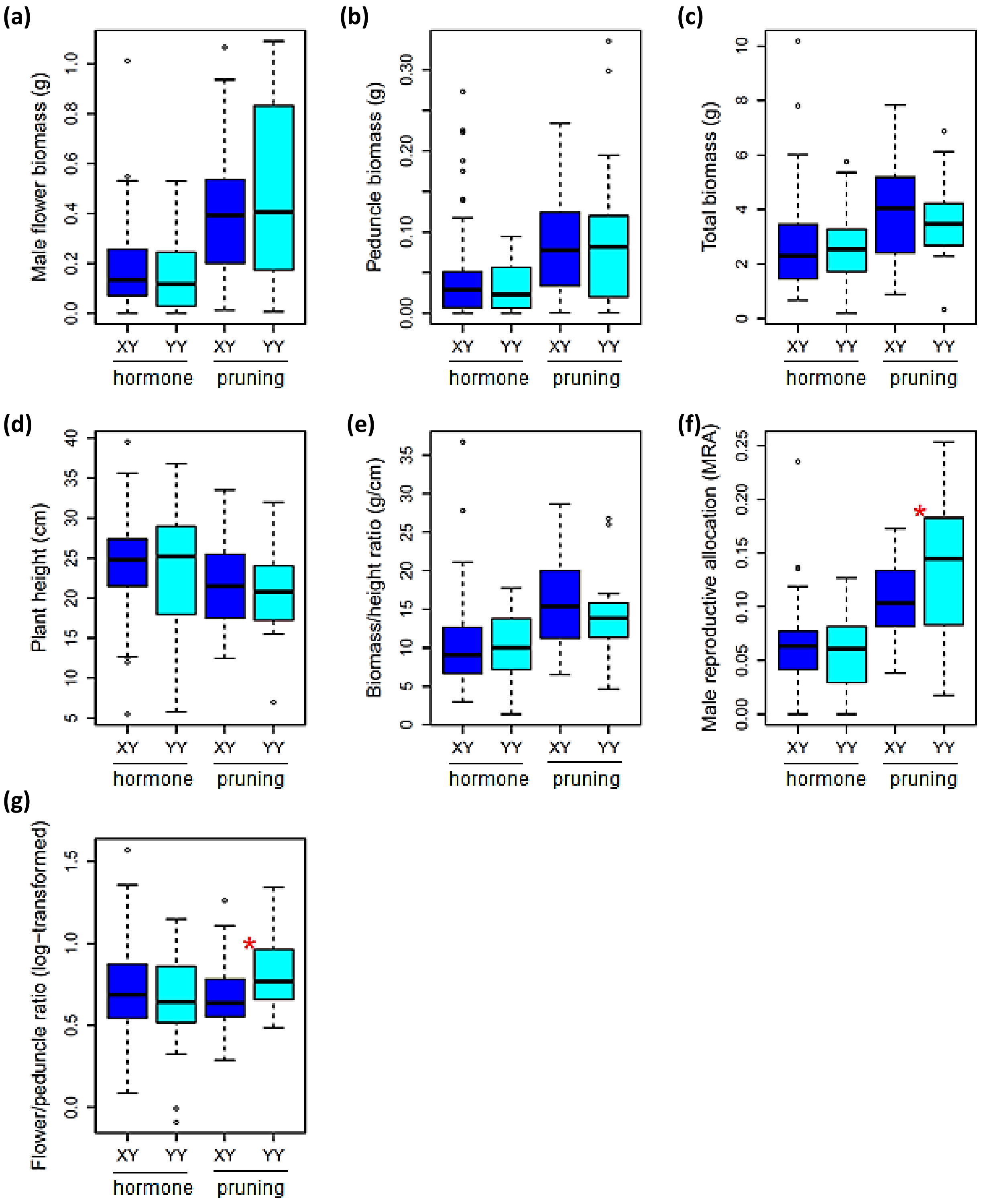

Figure S4 Summary of principal components analysis (PCA) for phenotypic measurements of F1 males from the hormone-induced experiment. Four independent traits are included for the analysis after normalisation: male flower biomass (flower_biomass), peduncle biomass (peduncle_biomass), total biomass (dry_biomass) and plant height (height).

(a) **(b)**

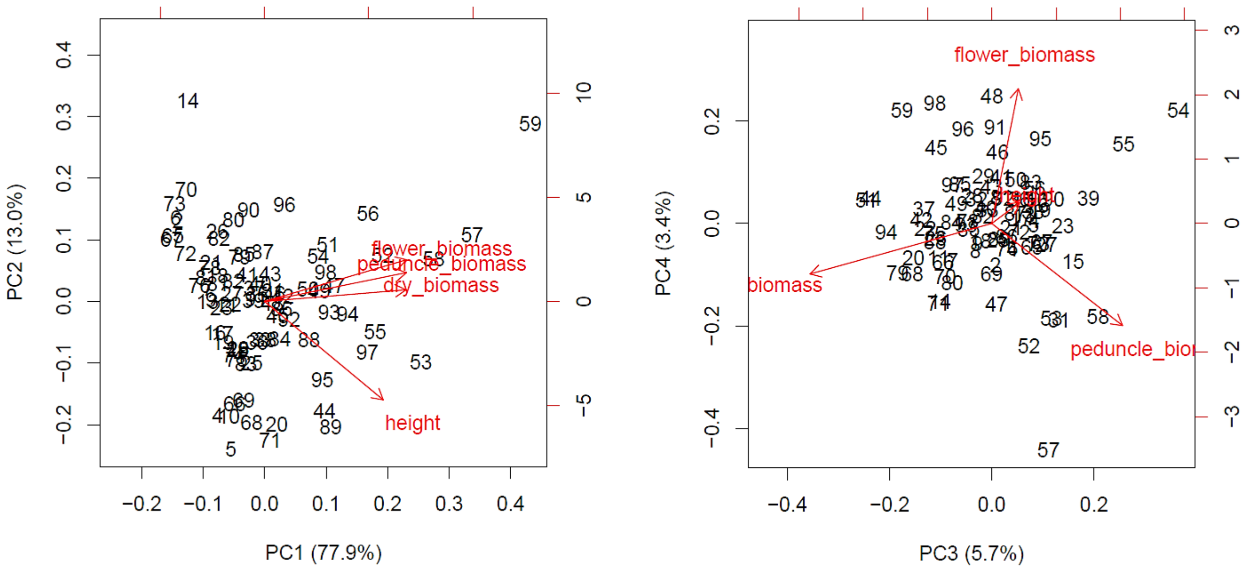

(c)

	PC1	PC2	PC3	PC4
male flower biomass	0.521	0.375	0.117	0.758
peduncle biomass	0.520	0.260	0.576	-0.575
total biomass	0.519	0.105	-0.800	-0.284
plant height	0.435	-0.884	0.124	0.119

Figure S5 Summary of PCA for phenotypic measurements of F1 males from the pruning-induced experiment.

(a)

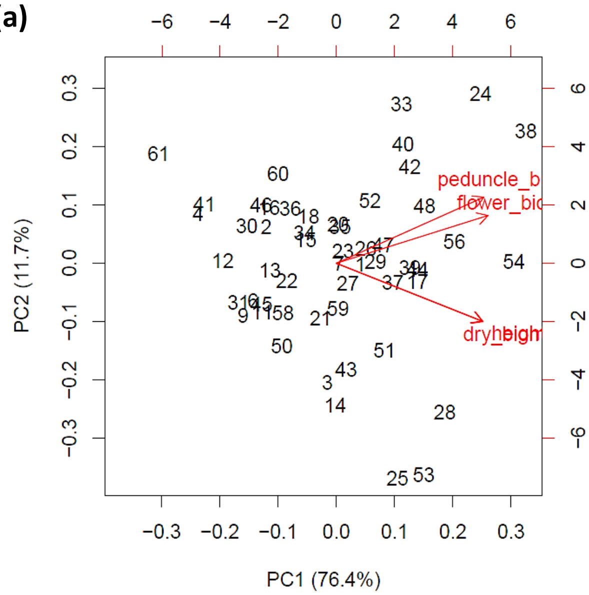

(b)

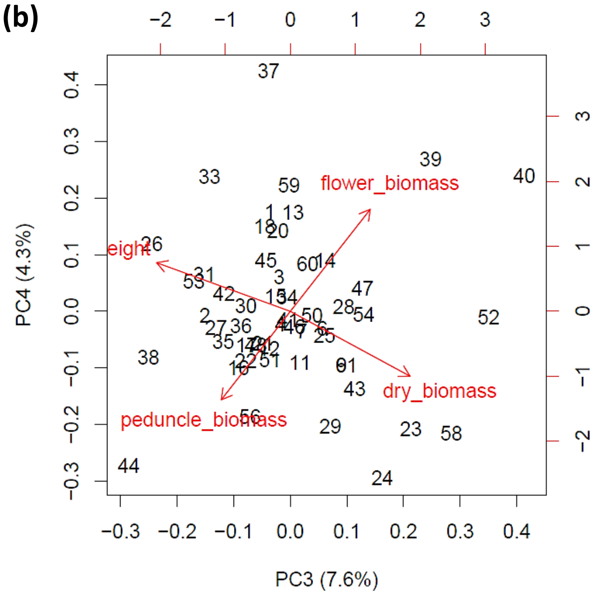

(c)

	PC1	PC2	PC3	PC4
male flower biomass	0.513	0.413	0.384	0.648
peduncle biomass	0.499	0.571	-0.332	-0.562
total biomass	0.496	-0.504	0.575	-0.412
plant height	0.493	-0.500	-0.642	0.309

Table S1 Summary of generalised linear mixed model on binomial genotype (XY versus YY) explained by the first 4 principal components of normalised independent phenotypic measurements. Left: male progeny from the hormone-induced experiment, with sampler and sampling date as random variables. Right: male progeny from the pruning-induced experiment, with family as a random variable. *P* values < 0.05 are in bold.

	Dependent Variables	
Predictors	genotype	
	Odds ratio (CI)	*p*
**Fixed Parts**		
(Intercept)	0.60 (0.38 – 0.94)	**0.026**
Comp.1	0.84 (0.62 – 1.12)	0.239
Comp.2	0.91 (0.49 – 1.69)	0.772
Comp.3	0.43 (0.16 – 1.20)	0.106
Comp.4	1.31 (0.33 – 5.19)	0.703
**Random Parts**		
$\tau_{00, date}$	0.000	
$\tau_{00, sampler}$	0.000	
N_{date}	3	
$N_{sampler}$	2	
ICC_{date}	0.000	
$ICC_{sampler}$	0.000	
Observations	86	
AIC	124.292	
Deviance	110.292	

	Dependent Variables	
Predictors	genotype	
	Odds ratio (CI)	*p*
**Fixed Parts**		
(Intercept)	0.36 (0.18 – 0.72)	**0.004**
Comp.1	1.06 (0.74 – 1.52)	0.744
Comp.2	3.44 (1.06 – 11.21)	**0.040**
Comp.3	0.85 (0.26 – 2.76)	0.783
Comp.4	4.19 (0.89 – 19.74)	0.070
**Random Parts**		
$\tau_{00, family}$	0.000	
N_{family}	19	
ICC_{family}	0.000	
Observations	53	
AIC	68.030	
Deviance	56.030	

Table S2 Pollen observation under electron microscopy. SEM, scanning electron microscopy; TEM, transmission electron microscopy. $N_{individuals}$, number of plants included for each sample type. N_{pollen}, number of pollen grains measured for size estimates under SEM. N_{normal}, number of anther samples that show normal pollen morphology. $N_{abnormal}$, number of anther samples with abnormal pollen morphology.

Sample type	$N_{individuals}$	Pollen size under SEM (µm)			TEM	
		N_{pollen}	Polar diameter (mean ± SE)	Equator diameter (mean ± SE)	N_{normal}	$N_{abnormal}$
Dried pollen from YY males	9	33	21.96 ± 3.64	17.52 ± 0.99	11	12
Dried pollen from XY males	9	36	22.70 ± 1.54	17.50 ± 1.18	9	2
Fresh pollen from XY males (control)	5	30	26.64 ± 1.16	15.23 ± 0.84	8	0

Methods S1 Identification between XY and YY males with a restriction enzyme-based assay based on sex-specific SNPs.

We used previously published genome capture sequencing data to identify one exon with male-specific SNPs across 10 populations spanning most of the species range (2 males and 2 females in each, see González-Martínez *et al.*, 2017 for details). We confirmed the presence of this SNP by direct Sanger sequencing of PCR products that amplify in both sexes in individuals from Barcelona (SP), Antalya (TR), Jerusalem (ISR), Southampton (UK), Trabzon (TR) and Volos (GR). The Sanger sequences (Figure **S5a**) were aligned using MAFFT (Katoh & Standley, 2013) in Geneious v9.1.5 (https://www.geneious.com). PCR amplification with primers 1911F (CGCTGTGACTGATCCTGGAA) and 1911R (CTCTTGAGATTGGCCTGCCA) at 55°C annealing temperature, 1 min extension at 72°C and 30 PCR cycles and resulted in a 727 bp product. The product contained two SNPs and could be reliably digested with restriction enzymes *BceAI* (X-specific) and *RseI* (Y-specific) separately, to result in an assay that distinguishes XY and YY individuals (Figure **S5b**).

González-Martínez SC, Ridout K, Pannell JR. **2017**. Range expansion compromises adaptive evolution in an outcrossing plant. *Current Biology* **27**: 2544–2551.e4.

Katoh K, Standley DM. **2013**. MAFFT multiple sequence alignment software version 7: improvements in performance and usability. *Molecular Biology and Evolution* **30**: 772–780.
